# Mitigating Global Oral Health Inequalities: Research Training Programs in Low- and Middle-Income Countries

**DOI:** 10.5334/aogh.3134

**Published:** 2020-11-05

**Authors:** Ana Lucia Seminario, Timothy DeRouen, Mimansa Cholera, Jennifer Liu, Prathip Phantumvanit, Arthur Kemoli, Jorge Castillo, Waranuch Pitiphat

**Affiliations:** 1 University of Washington, US; 2 Thammasat University, TH; 3 University of Nairobi, KE; 4 Universidad Peruana Cayetano Heredia, PE; 5 Khon Kaen University, TH

## Abstract

Although oral diseases are largely preventable, they are among the most non-communicable diseases globally, and they disproportionately burden disadvantaged communities, specially within low- and middle-income nations. The COVID-19 pandemic has highlighted the social, economic, and health inequalities in our society, including the existing global oral health inequalities. There is a shortage of dentist-scientist all around the world, especially in developing countries, such as Thailand. The National Institute of Dental and Craniofacial Research (NIDCR) and Fogarty International Center (FIC), joined efforts on creating research capacity in oral health in South East Asia through the Fogarty International Center Training Program in Clinical, Public Health and Behavioral Oral Health Research for Thailand (2006–2016). The University of Washington (USA), Thammasat University (Thailand) and Khon Kaen University (Thailand) partnered to conduct short-, medium- and long-term training programs to build regional oral health research capabilities. Investing in research has not only impacted trainees’ career development but enhanced advancement of oral health research of South East Asia. The success of partnership calls for expanding oral health research training in other low-income countries.

## The Problem

Oral diseases are among the most common non-communicable diseases worldwide [1]. According to the Global Burden of Disease Study, untreated dental caries in permanent teeth was the most prevalent disease among 313 assessed conditions, affecting more than 530 million children [2]. Oral diseases are not only highly prevalent, but access to care varies based on the country of residence. Socioeconomic inequalities in access to oral health services ranges from 35% in low-income countries to 60% in lower-middle-income countries, 75% in upper-middle income countries and 82% in high-income countries [3]. Gaps initiatives to prevent oral diseases and improve access to healthy environments require better dental public health systems, strong local oral health research capacity and a well-trained dental health workforce.

The number of dentists-scientists worldwide is decreasing as the new generation of dental students favor clinical paths [4]. Motivating young oral health professionals to either remain in academics (for junior faculty) or to dedicate their career to research (for students), needs to be supported by an infrastructure and environment that enables short, medium and long-term lines of research. Factors like the length of DDS-PhD training, high debt burden, economic disincentives as well as income differences between private and academic sectors are some of the major reasons for dental students to decline research paths. Because scientific and clinical training largely remain compartmentalized in dental school settings and poorly integrated within the institution at large [4], it is critical to invest in research education and in creating research infrastructure that generates robust research capacity for the next generation of oral health research professionals. The impact of investing in global health not only improves the quality of life of the community but has the direct potential of improving the economy at country level [4]. However, creating an oral health research site requires significant financial support that often is lacking in developing countries.

## Fogarty International Center and the National Institute of Dental and Craniofacial Research Have Successfully Partnered on Increasing Regional Research Capacity

The National Institutes of Health (NIH) has recognized the need to build the research training and capacity outside of the United States (U.S.) through the creation of the Fogarty International Center (FIC). FIC is dedicated to advancing the NIH mission by supporting and facilitating global health research conducted by the U.S. and international investigators, building partnerships between health research institutions in the U.S. and abroad, and training the next generation of scientists to address global health needs [5]. By facilitating exchanges among investigators, providing training opportunities and supporting promising research initiatives in developing countries, FIC has served as the bridge between NIH and the greater global health community for more than 50 years.

FIC efforts on increasing research training and capacity around the world include expanding training in policy development, implementation, and evaluation at the institutional, local, and national levels [6,7,8,9]. While the initial FIC support has traditionally been allocated to infectious diseases, new opportunities for noncommunicable diseases have been opened in partnership with other NIH institutes like the National Institute of Dental and Craniofacial Research (NIDCR). An example of such a partnership is found in Thailand.

### The South East Asia Model

The public health burden of oral diseases has influenced the development of Thai dental education. With almost 70 million inhabitants, the developing country of Thailand is an active member of the Association of South East Asian Nations interested in creating oral health research capacity and training. Since the first dental school opened in 1940, currently, there are now 12 public and 4 private dental schools offering the degree of Doctor of Dental Surgery (DDS) through 6 years of education with an additional 3 years of mandatory public service after graduating from public dental school [10]. While governmental efforts towards increasing clinical providers have been successful, there is a gap in the training and education required to create a cadre of dental researchers beyond clinical expertise, who would advance research that impacts public health.

For over 20 years, the University of Washington, School of Dentistry, has partnered with Thammasat University and Khon Kaen University to train a new generation of oral health researchers who have impacted the dental public health arena in South East Asia. As a result of this partnership, these institutions created the Fogarty International Center Training Program in Clinical, Public Health and Behavioral Oral Health Research for Thailand (D43 TW007768 and D43 TW009071, PI: DeRouen). By training over 200 trainees from 18 countries, this training site has brought Thailand to the international forefront of oral health research. As a result, Thai universities have become the regional resource for oral health research in South East Asia.

### Type of Trainings

#### Short-Term Workshops

Topics discussed in these 5-day workshops included general scientific principles in planning and carrying out clinical research projects, levels of evidence in evidence-based clinical research, study designs, principles in the design and conduct of randomized clinical trials, statistical concepts and principles in testing hypotheses, basic behavioral models and ethical issues in the conduct of research on human subjects, and research protocol writing.

#### Medium-Term Training

Junior faculty and graduate students from the two collaborating Thai institutions, as well as short-term trainees who attended previous workshops were eligible to participate in the Summer Institute in Clinical Dental Research Methods held at the University of Washington in Seattle, U.S. In addition to clinical/public health/behavioral pathways, program participants included those pursuing advanced basic sciences research and degrees. This six-week program was designed to provide a short but intensive research training program for dental school faculty and professionals interested in clinical research. Specifically, core courses included biostatistics, clinical epidemiology and study design, behavioral research in dentistry, a data analysis workshop, a short course in randomized clinical trials, and a seminar on grantsmanship.

#### Long-Term Training

The third level of training was designed to augment and strengthen oral health research pathways in existing Ph.D. programs in Oral Science at Thammasat and Khon Kaen Universities. Trainees with English language skills spent one year in Seattle at the University of Washington to accomplish following three things: a) undertake the coursework in biostatistics, epidemiology or health services at the UW School of Public Health, that they might not have been able to access in Thailand. The kinds of coursework trainees would undertake depended on their interests, but were likely to be biostatistics courses involving design of medical studies, survival analysis, logistic regression, and analysis of correlated data; epidemiology courses on genetic epidemiology, exposure measurement, nutritional epidemiology, clinical epidemiology; and health services courses on applied community research, health program evaluation, health promotion and disease prevention, and evaluation of health program costs and outcomes; b) during the one-year stay in Seattle, trainees worked with one or more of the UW mentors on research projects they were developing to enhance their learning experiences; and c) trainees were required to plan and finalize a dissertation research project that they would work on over the period of the next year in Thailand.

#### Former Trainees’ Perspectives

**Figure 1:**
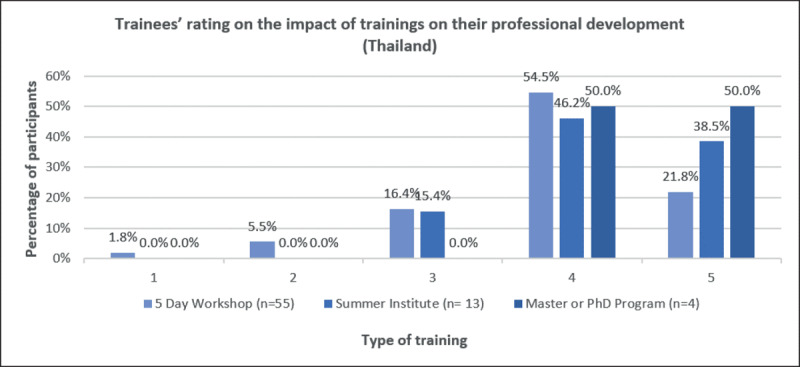
Trainees’ rating on the impact of trainings on their professional development.

**Figure 2:**
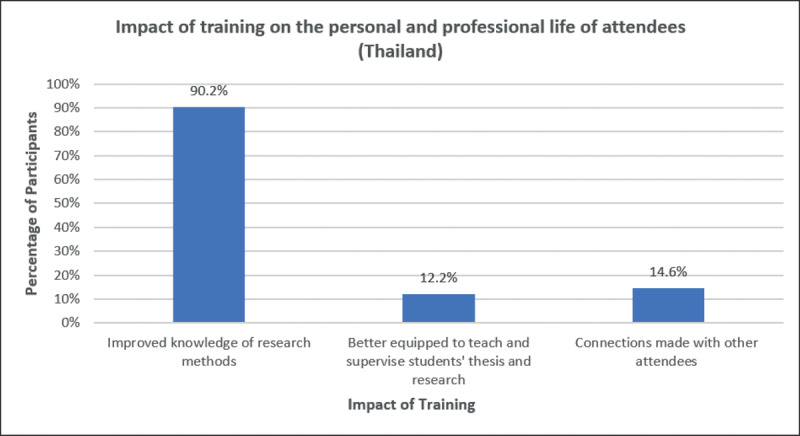
Impact of training on the personal and professional life of attendees.

With the purpose to provide a perspective on the impact of the FIC and NIDCR support on creating training and research capacity in South East Asia, we conducted a survey of former trainees to inform us on their perception on how the Fogarty International Center Training Program in Clinical, Public Health and Behavioral Oral Health Research for Thailand impacted their professional paths. All the training types were rated high on a scale of 0–5 by the trainees (Figure 1). Out of the 74 responses (47.1%), more than half (59.5%) of the survey respondents were female participants while the largest proportion of responses (43.2%) belonged to the 40–49 years age group. The impact of NIH support expanded to a diverse group of academicians from South East Asia. Besides Thailand, participants originated from a diverse geographical pool which included Nepal, Japan, Myanmar, Vietnam, Mongolia, Indonesia, India, Bhutan, Brazil, Cambodia, China, Malaysia, Philippines, and New Zealand. Most participants (74.3%) were enrolled in short-term training, 17.6% enrolled in medium-term training and 5.4% enrolled in long-term training. From the participants with PhD (or currently enrolled in a PhD program) as their highest level of education, the majority had their degrees from their own countries of origin (other than Thailand). The perceived impact on how these training types influenced trainees at personal and at professional levels was assessed through open-ended question. Three major themes emerged, and responses were not exclusive: a) Improved knowledge of research methods (90.2%); b) Better equipped to teach and supervise students’ thesis and research (12.2%); and c) Connections made with other attendees (14.6%) (Figure 2). Regardless of the rigorous setup and the fact that the trainings were in English (second language for trainees), fellows not only adapted but succeeded on fully completing their trainings and advanced academically in their home institutions.

## Advocating for More Opportunities to Enhance Global Oral Health

### Initial Efforts in Peru

In Peru, the past three decades have witnessed impressive growth in biomedical research catalyzed from efforts made among local educational institutions and NIH securing international partnerships and funding through research training programs [11]. As a result, Peruvian physician-scientists have built and sustained longstanding international partnerships with funding accelerating quality research on diseases of local importance. While oral health is not among the topics funded for creating research training and capacity, first steps have been conducted due to inter-professional partnership with non-dental researchers. Specifically, there have been two workshops in dental research methods from NIH supplement grants (in 2014 5D43TW007768-05 PI: DeRouen; and in 2015, Northern/Pacific Global Health Research Fellows Training Consortium 2015 5R25TW009345 PI: Zunt). The workshops were conducted in Lima. In the 5-day 2014 and 2015 workshops, dental junior faculty participated from various geographical areas within Peru, Spain and from other Latin American countries (Colombia, Argentina, Chile, Venezuela, and Argentina). Participants practiced various specialties, including orthodontics, pediatric dentistry, endodontics, implantology, community dentistry, special care needs, esthetics, prosthodontics, and periodontics. For both workshops, the consensus on the evaluations conducted was that the content was highly relevant, delivered at the right level, and could be utilized at their home institutions.

### Kenya as a Promising Site for Global Oral Health Research Training

Kenya is a lower middle-income Sub-Saharan country in Africa with a population of more than 48 million people. People living in poverty (36.1% of the population) have impaired overall health and are at high risk for HIV infection [12,13]. With 25,000 deaths estimated in 2018, HIV/AIDS is still estimated to cause a high proportion of deaths annually [14]. Across Kenya, the burden of oral diseases is high. The oral health care providers in Kenya are overstretched by the large population they serve, and most of these dentists are based in large urban centers, leaving large segments of the population in rural areas underserved [15]. According to the 2015 National Oral Health Survey, prevalence of dental caries among children was 23.9% and 34.3% among adults [15]. Approximately 75% of the children surveyed and nearly all the adult population surveyed (98.1%) had signs of periodontal disease, an oral condition that affects the gums and bones [15]. Strengthening oral health research capacity and the resultant improvement in the oral health related quality of life cannot be over-emphasized when considering the broader general health goal. The Global Forum for Health Research acknowledges that Africa needs to scale up her research agenda [16]. Due to the current Kenyan HIV research training and capacity, oral health has the unique opportunity to leverage on existing resources, platforms, and collaborations. Capacity building of the existing Kenyan workforce through multidisciplinary approach would result in production of oral health researchers who would leverage the technological advancements for the benefit of the population especially in resource limited settings [17]. While in the developed world scientific and technological advances in diagnosis, treatment and prevention strategies may be engrained in the core-curricula in the dental schools, this may not be the case in the dental schools in Kenya. Investing in oral health research training and capacity will enhance HIV research, broad multidisciplinary collaboration and strengthen Kenyan HIV public health efforts.

## Conclusion

We aimed to provide opportunities for decreasing global oral health inequalities by investing in creating research training and capacity building in low- and middle-income countries. To this date, only one such training site has been funded by FIC targeting oral health. We need to increase our advocacy efforts to expand oral health within FIC and global health within NIDCR. With this article, we hope to provide insights on the effects of long-term investments and efforts made in building research capacity in Thailand, and to advocate to FIC and NIDCR to expand such opportunities to other low-middle-income countries.

## References

[1] World Health Organization. Who.int. https://www.who.int/news-room/fact-sheets/detail/oral-health (Accessed September 1, 2020).

[2] GBD 2017 Disease and Injury Incidence and Prevalence Collaborators. Global, regional, and national incidence, prevalence, and years lived with disability for 354 diseases and injuries for 195 countries and territories, 1990–2017: A systematic analysis for the Global Burden of Disease Study 2017 [published correction appears in Lancet. 2019 June 22; 393(10190): e44]. Lancet. 2018; 392(10159): 1789–1858. DOI: 10.1016/S0140-6736(18)32279-7 30496104 PMC6227754

[3] Hosseinpoor AR, Itani L, Petersen PE. Socio-economic inequality in oral healthcare coverage: Results from the World Health Survey. J Dent Res. 2012; 91(3): 275–281. DOI: 10.1177/0022034511432341 22205634

[4] D’Souza RN, Colombo JS. How research training will shape the future of dental, oral, and Craniofacial Research. J Dent Educ. 2017; 81(9): eS73–eS82. DOI: 10.21815/JDE.017.037 28864807

[5] Fogarty International Center. Our global health research mission and vision – Fogarty international center @ NIH. Nih.gov. https://www.fic.nih.gov/About/Pages/mission-vision.aspx (Accessed September 23, 2020).

[6] Kass NE, Ali J, Hallez K, Hyder AA. Bioethics training programmes for Africa: Evaluating professional and bioethics-related achievements of African trainees after a decade of Fogarty NIH investment. BMJ Open. 2016; 6(9): e012758 DOI: 10.1136/bmjopen-2016-012758 PMC5030587 27633644

[7] Ramirez AG, Gallion KJ, Perez A, Adeigbe RT, Munoz E, Pasick RJ. Éxito!: Making an impact in training Latinos for doctorates and cancer research. J Cancer Educ. 2019; 34(5): 928–937. DOI: 10.1007/s13187-018-1397-6 30014170 PMC6335193

[8] Strosberg MA, Gefenas E, Loue S, Philpott S. Building research ethics capacity in post-communist countries: Experience of two Fogarty training programs. J Empir Res Hum Res Ethics. 2013; 8(5): 28–39. DOI: 10.1525/jer.2013.8.5.28 24384514 PMC4259573

[9] Saenz C, Heitman E, Luna F, Litewka S, Goodman KW, Macklin R. Twelve years of Fogarty-funded bioethics training in Latin America and the Caribbean: Achievements and challenges. J Empir Res Hum Res Ethics. 2014; 9(2): 80–91. DOI: 10.1525/jer.2014.9.2.80 24782074 PMC5523831

[10] Vivatbutsiri P, Iempook T, Wonghinkong S, Sopa S, Detsomboonrat P. Dental school tracks related to the retention of dentists in Thai government service: A cross-sectional survey. Hum Resour Health. 2020; 18(1): 5 DOI: 10.1186/s12960-020-0444-7 31992321 PMC6988324

[11] Belter CW, Garcia PJ, Livinski AA, Leon-Velarde F, Weymouth KH, Glass RI. The catalytic role of a research university and international partnerships in building research capacity in Peru: A bibliometric analysis. PLoS Negl Trop Dis. 2019; 13(7): e0007483 DOI: 10.1371/journal.pntd.0007483 31306424 PMC6658117

[12] World Bank Group. Policy Options to Advance the Big 4. http://documents.worldbank.org/curated/en/327691523276540220/pdf/125056-WP-P162368-PUBLIC-KenyaEconomicUpdateFINAL.pdf (Accessed September 1, 2020).

[13] World Health Organization. Who Country Cooperation Strategy Kenya. Medium – Term Support Strategy 2014–2019. https://www.afro.who.int/sites/default/files/2017-05/who-kenya-country-cooperation-strategy-2014_2019.pdf (Accessed September 1, 2020).

[14] UNAIDS. https://www.unaids.org/en/regionscountries/countries/kenya (Accessed September 1, 2020).

[15] Ministry of Health. Kenya national oral health survey report. 2015 https://profiles.uonbi.ac.ke/gathece/files/kenya_national_oral_health_survey_report_2015.pdf (Accessed September 1, 2020).

[16] Naidoo S, Dimba E, Yengopal V, Folayan MO, Akpata ES. Strategies for oral health research in Africa and the middle eastern region. Adv Dent Res. 2015; 27(1): 43–49. DOI: 10.1177/0022034515575539 26101339

[17] Mumghamba EG, Joury E, Fatusi O, Ober-Oluoch J, Onigbanjo RJ, Honkala S. Capacity building and financing oral health in the African and Middle East region. Adv Dent Res. 2015; 27(1): 32–42. DOI: 10.1177/0022034515578909 26101338

